# The development of multiplex PCR assays for the rapid identification of multiple *Saccostrea* species, and their practical applications in restoration and aquaculture

**DOI:** 10.1186/s12862-024-02250-1

**Published:** 2024-05-21

**Authors:** Marina A. Richardson, Nikolina Nenadic, Max Wingfield, Carmel McDougall

**Affiliations:** 1 Coastal and Marine Research Centre, Australian Rivers Institute, School of Environment and Science, Griffith University, Nathan, Queensland Australia; 2 Department of Agriculture and Fisheries, Queensland Government, Woorim, Queensland Australia; 3 Scottish Oceans Institute, University of St Andrews, St Andrews, United Kingdom

**Keywords:** *Saccostrea*, Aquaculture, Restoration, Oyster, Oyster reef, Oyster reef restoration, Molecular, Species-specific PCR, Tropical oysters

## Abstract

**Background:**

The ecology and biology of oysters (Ostreidae) across the tropics is poorly understood. Morphological plasticity and shared characteristics among oysters have resulted in the misidentification of species, creating challenges for understanding basic species-specific biological information that is required for restoration and aquaculture. Genetic barcoding has proven essential for accurate species identification and understanding species geographic ranges. To reduce the costs of molecular species identification we developed multiplex assays using the cytochrome c oxidase subunit I (COI or cox1) barcoding gene for the rapid identification of five species of oysters within the genus *Saccostrea* that are commonly found in Queensland, Australia: *Saccostrea glomerata*, *Saccostrea* lineage B, *Saccostrea* lineage F, *Saccostrea* lineage G, and *Saccostrea spathulata* (lineage J).

**Results:**

Multiplex assays were successful in species-specific amplification of targeted species. The practical application of these primers was tested on wild spat collected from a pilot restoration project in Moreton Bay, Queensland, with identified species (*S. glomerata*, lineage B and lineage G) validated by Sanger sequencing. DNA sampling by extraction of oyster pallial fluid was also tested on adult oysters collected from the Noosa estuary in Queensland to assess whether oysters were able to be identified non-destructively. DNA concentrations as low as 1 ng/ μL still amplified in most cases, allowing for identification, and mortality at 6 weeks post pallial fluid collection was low (3 out of 104 sampled oysters).

**Conclusion:**

These multiplex assays will be essential tools for species identification in future studies, and we successfully demonstrate their practical application in both restoration and aquaculture contexts in Queensland. The multiplex assays developed in this study outline easily replicable methods for the development of additional species-specific primer sets for the rapid identification of other species of *Saccostrea* found across the Indo-Pacific, which will be instrumental in unravelling the taxonomic ambiguities within this genus in tropical regions.

**Supplementary Information:**

The online version contains supplementary material available at 10.1186/s12862-024-02250-1.

## Background

The global decline of oyster reefs is unprecedented compared to other intertidal estuarine marine habitats, and they are now considered functionally extinct in many regions [1]. There is a widely recognised need to restore oyster reefs and the ecosystem services they once provided, including water filtration, nutrient cycling, shoreline protection, and habitat for fish and marine invertebrates [2–5].

Globally, tropical regions contain over four times more reef-building species of oysters than temperate regions [6]. Molecular techniques are required for accurate identification, however, as shell morphology is highly variable which has resulted in species being visually misidentified [7, 8]. Misidentification is especially prevalent within the genus *Saccostrea*, where multiple species occur across Australia and the Indo-Pacific. Multiple species within this genus have been collectively lumped into two “superspecies”; *Saccostrea scyphophilla* (previously *Saccostrea mordax*) which is comprised of at least three genetically distinct lineages, designated A – C, and *Saccostrea cuccullata* which contains at least ten genetically distinct lineages (designated A – J), with most lineages likely representing a separate species [7, 9–12]. For example, *Saccostrea scyphophilla* lineage C has now been described as a new species *Saccostrea mordoides* [13], *Saccostrea cuccullata* lineage J has recently been assigned to the species *Saccostrea spathulata* [12], *Saccostrea cuccullata* lineage F may correspond to the species *Saccostrea malabonensis* [10], and accidental sampling of *Saccostrea cuccullata* lineage B and G during a *Saccostrea glomerata* population study showed that these three lineages are likely separate species through nuclear DNA markers [14].

Tropical Australia has been identified as having high potential for the establishment and expansion of oyster aquaculture and oyster reef restoration, however the high diversity of species and taxonomic uncertainties pose a challenge [6, 12, 15]. For example, *Saccostrea echinata* is a small spiky oyster endemic to the Indo-Pacific and likely Australia [7, 11, 16], however, the name continues to be incorrectly applied to the tropical blacklip oyster (now known to be *S. spathulata* [12]), a promising species for aquaculture in the Australian tropics [17–20]. The use of “superspecies” names in scientific literature, especially *S. cuccullata*, is also problematic as it is unclear which specific lineage research is reporting on [21–24]. The past use of what we now know to be incorrect or “superspecies” names has trickled through to government and industry, causing difficulty for summarising species-specific biological information necessary for advancing restoration and aquaculture [6]. There is a need for all stakeholders to adopt the lineage naming system proposed by Lam and Morton, 2006 to delineate species and species-specific information until species boundaries have been ascertained and scientific names can be assigned [9, 10, 12, 15].

Molecular research has been essential in the accurate identification of morphologically similar oyster species and resolving taxonomic uncertainties. The cost of molecular work can present a barrier to research, however, the development of tools such as species-specific primers can drastically reduce costs by allowing identification by gel electrophoresis rather than nucleotide sequencing. For example, in China species-specific primer sets were developed using the mitochondrial cytochrome oxidase I (COI) gene for use within a multiplex PCR for the identification of five co-occuring oysters within the genus *Magallana* (previously *Crassostrea*): *Magallana angulata*, *Magallana ariakensis*, *Magallana gigas, Magallana hongkongensis* and *Magallana sikamea* [25]. The development of species-specific primers can also allow for the analysis of larger sample sizes for ecological studies. Primer sets using the mitochondrial ND5 gene were developed for the identification of *M. sikamea* and *M. ariakensis* which are known to create mixed species oyster reefs in China [26]. The development of these primer sets facilitated research that explored settlement preferences between the two species and highlighted potential reasons for limited restoration success [27].

While most lineages within the *S. cucullata* complex are yet to be assigned to a new or existing species, molecular work has enabled a better understanding of habitat preferences and geographic ranges which will aid in species identification and understanding aquaculture and restoration potential in localised regions [7, 9, 15, 28]. Five species belonging to the *S. cuccullata* complex have recently been documented in the Queensland tropics: lineage B, lineage F, lineage G, lineage I and *Saccostrea spathulata* (lineage J) [12]. Most of these species are sympatric with one another as well as with *S. glomerata* (in subtropical locations), and in most cases are unable to be distinguished from one another morphologically. To enable the rapid identification of these species we developed species-specific primers for use in multiplex PCRs and gel electrophoresis and demonstrate their practical applications in both restoration and aquaculture.

## Methods

### Primer design

The species chosen for primer development were *S. glomerata*, lineage B, lineage F, lineage G and *S. spathulata*. Lineage I was not selected as this species has several distinctive characteristics including broad but delicate purple lobe-like squamae (lamellae) on the lower/bottom (left) valve, internal shell iridescence, and preferred settlement on the roots of mangroves, enabling straightforward identification [12, 16]. *S. scyphophilla* and *S. mordoides* were also excluded as these oysters are not part of the *S. cuccullata* complex and can be distinguished morphologically by their distinctive shape, deep longitudinal ridges, and purple colouration [12].

**Fig. 1:**
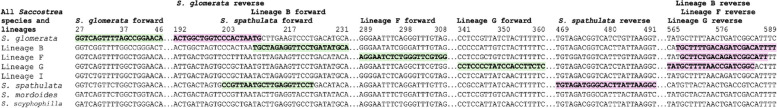
Nucleotide sequences among all species and lineages at primer-annealing regions. Forward primers are highlighted in green and reverse primers are highlighted in purple

The cytochrome c oxidase subunit I (COI or cox1) barcoding gene was chosen as the target for primer design based on the availability of sequences for all species of interest, and the presence of sufficient nucleotide diversity to enable the design of species-specific primers. All publicly submitted COI gene sequences from Australia and the Indo-Pacific were compiled for each target species from NCBI (https://www.ncbi.nlm.nih.gov), and one sequence representing the consensus was used for primer design. Several primer sets were designed for each species using Primer3 (https://primer3.ut.ee) specifying the following parameters: optimal primer size of 20 base pairs (minimum 18 and maximum 23); optimal annealing temperature of 59 °C (minimum 57 °C and maximum 62 °C); and 50 % optimal GC content (minimum 30 % and maximum 70 %). Final primers were then selected based on the uniqueness of nucleotide positions compared to the same gene regions of other species, to ensure that polymorphic sites within species were avoided, and to ensure that there was a minimum difference of 55 base pairs between the product size of each species for readability in gel electrophoresis. Details of species-specific primer sets, product sizes and sequence of origin for primer design can be found in Table 1. The number of unique nucleotide positions between each species/lineage at each region of all species-specific forward and reverse primers can be found in Table 2. Nucleotide sequences among all species and lineages at primer-annealing regions can be found in Fig. 1.

**Table 1:**
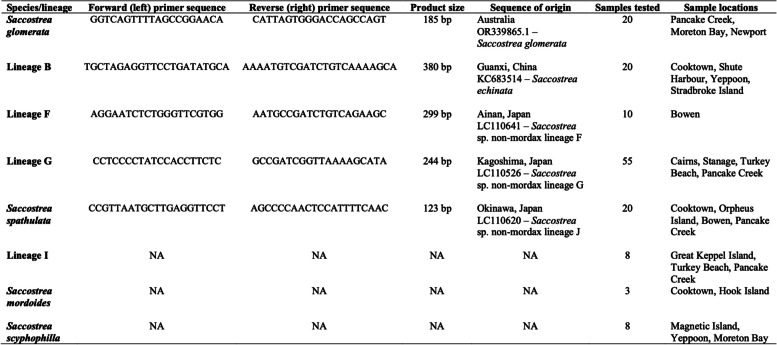
Summary of primers designed for morphologically similar Saccostrea species in Queensland

**Table 2:**
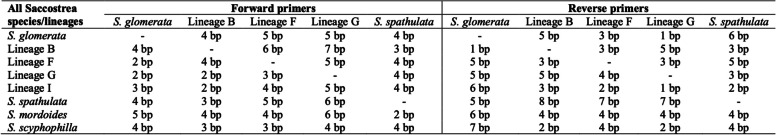
The number of unique nucleotide positions between each species/lineage at each region of species-specific forward and reverse primers

Primers were tested on oyster DNA from specimens collected across Queensland that had previously been unambiguously identified by 16S and/or COI barcoding [12]. Species-specific primer sets were first tested individually on confirmed DNA from 20 individuals of the target species to confirm functionality, except for lineage F where only 10 samples were used due to low sample availability. In each reaction the following quantities were added to the PCR mixture; 2 μL of New England Biolabs ThermoPol® 10X buffer (Catalog # B9004); 1 μL of each primer (forward and reverse) at a concentration of 20 μM; 0.4 μL of New England Biolabs® Deoxynucleotide (dNTP) Solution Mix (Catalog #N0447) at a concentration of 10 mM; and 0.2 μL of New England Biolabs® Taq DNA polymerase (Catalog # M0267). All DNA used for testing was added at a concentration of 25 – 50 ng/ μL, and PCR mixtures were then brought to 20 μL with molecular grade water. A Bio-Rad T-100™ thermal cycler was used for PCR reactions and the thermoprofile consisted of 5 min at 94 °C as initial denaturation, followed by 25 cycles of 30 sec at 94 °C for denaturation, 30 sec at 62 °C for annealing, 30 sec at 68 °C for extension, and final extension at 68°C for 5 min. 10 μL of each PCR reaction was then visualised by gel electrophoresis at 110 V for 30 minutes in a 2.5 % TAE agarose gel with a 100 bp DNA ladder.

We then tested each primer set on confirmed DNA from the seven remaining *Saccostrea* lineages and species found in Queensland (including lineage I, *S. scyphophilla* and *S. mordoides*) to check for amplification of non-targeted species using the same thermoprofile. Where required, gradient PCRs (with annealing temperatures ranging from 62 – 70 °C) were conducted for further optimisation. Once primers were optimized to amplify targeted species, we tested all five primer pairs together in a single multiplex PCR. Unfortunately, successful multiplex PCR of all five primers was unable to be achieved as this decreased the positive detection rate of all species. Instead, four-species multiplex assays using all combinations of primer sets using the same initial thermoprofile were successful.

### Practical applications – restoration

**Fig. 2:**
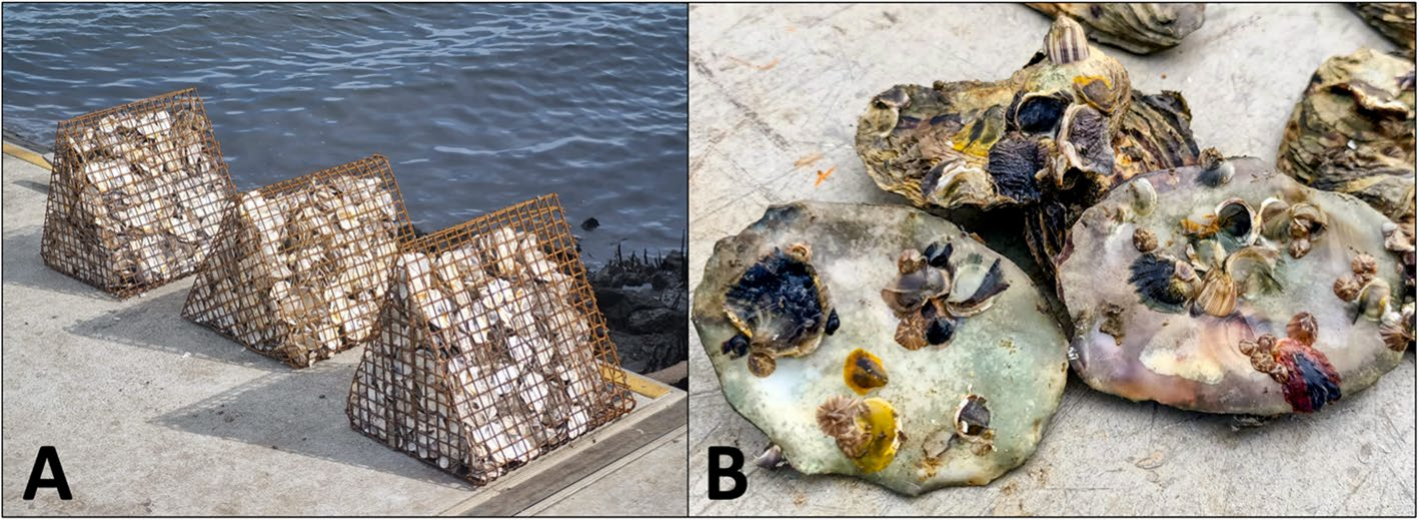
**A** Gabion baskets designed and used by OzFish for oyster reef restoration in southeast Queensland; and **B** an example of different spat morphologies recruiting to oyster shell in gabion baskets. Photo credit: Robert Porter

Gabion baskets filled with sterilised oyster shell are becoming an increasingly popular method of oyster reef restoration in the US [29, 30]. This method has now been adopted in Queensland as a part of OzFish Unlimited’s “Shellfish Revolution” restoration initiative (see https://ozfish.org.au/programs/shellfish-restoration/). To test our primers on wild oysters recruited in a restoration pilot project, we deployed 15 gabion baskets built by OzFish (Fig. 2A) at a commercial oyster lease in Moreton Bay, Queensland (-27° 27’ 31.9644” N and 153° 23’ 45.4236” S) in November 2022 to coincide with known *S. glomerata* spawning season [31]. To account for other species of *Saccostrea* that may have had different spawning triggers/seasons [32–34], baskets were left to establish for one year. In November 2023, five spat per basket (*n* = 75) were collected for identification, with care taken to sample a broad range of morphologies and sizes (Fig. 2B).

To extract DNA to enable species identifications to be made, a piece of mantle tissue was sampled from spat over 2 cm in size and all tissue was collected for smaller spat. Tissue samples were placed in sterilised microcentrifuge tubes upon collection and stored in 70% ethanol for later analysis. DNA was then extracted using the DNeasy Blood and Tissue Kit (Qiagen), and yields were measured in a Qubit Fluorometer and diluted to concentrations of 20 – 50 ng/ μL.

Although *S. glomerata* is the recognised reef-building species in southeast Queensland, lineage B and lineage G are also known to occur in this region [12, 14]. Lineage B, lineage G and *S. glomerata* primer sets were therefore selected for use in a multiplex PCR for species identification. DNA was amplified in a multiplex PCR using the same PCR mixture and thermoprofile described above, and 10 μL of PCR product was then visualised via agarose gel electrophoresis. Although lineage F and lineage J do not have known distributions in Moreton Bay [12], all unamplified DNA was run in a multiplex PCR using these primer sets as a precaution, however no DNA amplified. A subset of samples from each identified species as well as all unidentified samples were then amplified in a PCR using 16S ribosomal RNA (16S) primer sets described in McDougall and Walker, 2021 [35] and sent to Macrogen for Sanger sequencing to validate results.

### Practical applications – aquaculture

Tissue sampling from oysters for the purpose of DNA extractions often results in mortality. This is problematic for many applications, including aquaculture which relies on the identification of live specimens for future hatchery production. Lineage G is a strong candidate for aquaculture given its morphological similarity to *S. glomerata* and widespread distribution across Australia and the Indo-Pacific [12]. To positively identify live lineage G specimens, we collected 20 oysters from the Noosa estuary in southeast Queensland (-26° 23’ 37.3776”N and 153° 2’ 30.6888” E) where populations of lineage G and *S. glomerata* are found [12]. To non-destructively sample DNA, we first trialled magnesium chloride (MgCl_2_) baths following the methods of Nowland et al., 2021. We had limited success obtaining DNA samples with this method which we attribute to the small size of our collected oysters compared to the tropical blacklip.

To trial a second method, 104 oysters were collected from the same estuary and transported to the Bribie Island Research Centre. A mechanical handheld 1 mm drill was used to make a small hole in the ventral half of the right (upper) valve, with care to avoid contact with the animal inside. A 23G 0.65x32mm needle on a 1ml syringe was inserted and 250 – 500 μL of pallial fluid was extracted, placed in a microcentrifuge tube and stored at 4 °C until processing. The hole was then plugged with beeswax and covered with quick dry waterproof superglue, and the oyster retained in aquaria at the facility to monitor recovery.

Prior to DNA extractions, microcentrifuge tubes containing pallial fluid were spun down for 5 minutes at 13,000 rpm to create a pellet. Due to expected low DNA yields from pallial fluid, the DNeasy Blood and Tissue Kit (Qiagen) protocol was modified as DNA binding in silica-solution versus silica spin columns can increase DNA yields [36]. In summary, 180 μL of Buffer ATL (Qiagen) and 10 μL of ProK buffer was added to the pallial fluid and incubated at 56 °C for 3 – 6 hours. 400 μL of Buffer AL was added to the solution and vortexed for 15 seconds. 400 μL of 100% ethanol was added to the solution and vortexed for 15 seconds, followed by incubation at room temperature for 5 minutes. 20 μL of silica solution was added, mixed, and incubated at 50 °C for 15 minutes. The solution was then centrifuged for 30 seconds at 13,000 rpm, and supernatant was discarded. 500 μL of Buffer AW1 (Qiagen) was added and the silica pellet was resuspended. The solution was centrifuged at 13,000 rpm for 30 seconds, supernatant was discarded, and the previous wash step was repeated with Buffer AW2 (Qiagen). Once supernatant from the AW2 wash step was discarded, the silica pellet was left to air dry for 5 minutes. The silica pellet was then resuspended in 10 μL of H_2_O and centrifuged at 500 rpm for 2 minutes. DNA yields were measured in a Qubit Fluorometer and ranged from approximately 15 ng/ μL to 50 ng/ μL, however some samples were less than 1 ng/ μL. Because *S. glomerata* and lineage G were the only species of interest for this experiment, these were the only primers used in the multiplex PCR.

## Results

**Fig. 3:**
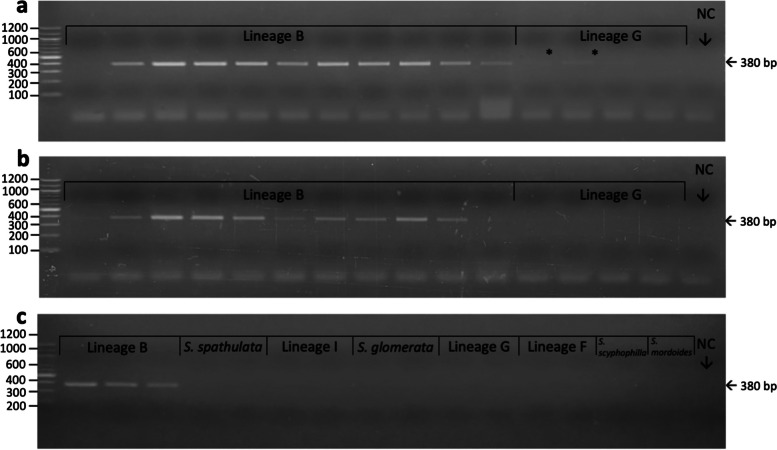
Gel electrophoresis of PCR products using the lineage B primer set (380 bp): **a** lineage B and lineage G DNA amplified at an annealing temperature of 68 °C (* denotes non-targeted amplification of lineage G DNA); **b** lineage B and lineage G DNA amplified at an annealing temperature of 70 °C; and **c** PCR products of all Queensland *Saccostrea* species DNA amplified at an annealing temperature of 70 °C (lineage G DNA amplified in this PCR had previously produced non-targeted bands). NC = negative control. See Supplementary Figures S1 – S3 for original photographs

All species-specific primer pairs successfully and exclusively amplified targeted species with the exception of the lineage B primer set, which amplified a product of the same expected size from all lineage I DNA. Six alternative lineage B primer sets were designed and tested but none showed species specificity. A gradient PCR was then performed from 62 °C – 68 °C using the original primer set to determine the optimal annealing temperature for species specificity; this was achieved with an annealing temperature of 68°C. Occasional non-specific amplification of lineage G samples was also observed with the lineage B primers at the expected size for lineage B products (four out of 55 tested lineage G DNA samples). We repeated the gradient PCR, however, two bands were still faintly present at 68 °C (Fig. 3a). This was resolved by raising the annealing temperature to 70 °C, however this decreased the positive detection rate of the Lineage B primers (Fig. 3b). Species specificity of the Lineage B primer sets is shown in Fig. 3c.

**Fig. 4:**
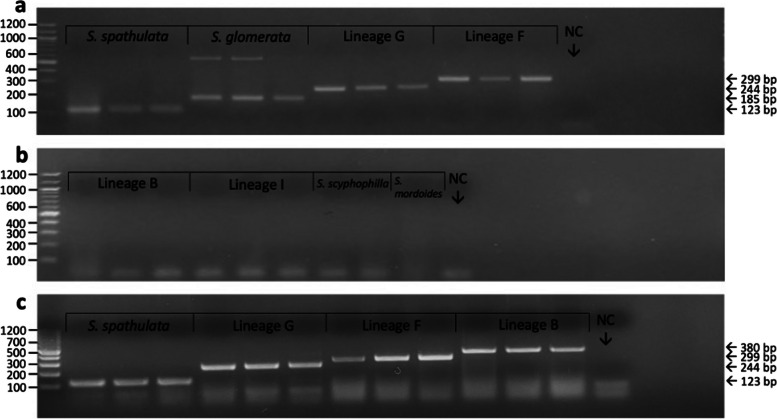
Gel electrophoresis of PCR products demonstrating species-specific amplification of targeted species: **a** Saccostrea multiplex 1 PCR products of targeted *Saccostrea* species DNA amplified at an annealing temperature of 62 °C; **b** Saccostrea multiplex 1 PCR products of non-targeted *Saccostrea* species DNA (same PCR as 3a but run in a different gel); and **c** Saccostrea multiplex 2 PCR products of targeted *Saccostrea* species DNA amplified at an annealing temperature of 62 °C. NC = negative control. See Supplementary Figures S4 – S6 for original photographs

Four-species multiplex assays using all combinations of primer sets were successful in species-specific amplification of targeted species (Fig. 4). Although a single five-species multiplex assay was unable to be achieved, *S. glomerata* has a subtropical distribution and does not overlap with lineage F and has very little overlap with *S. spathulata*. It is therefore highly unlikely that a five-species multiplex assay would be required for species identification in Queensland. The *S. glomerata* primer showed some non-specific amplification, producing both the expected product at 185 bp, as well as a larger band of approximately 600 bp (Fig. 4a). This non-specific amplification will not impact species identification as the largest product size for primers designed in this study is 380 bp.

### Spat identification

Three species of *Saccostrea* were identified from gabion baskets deployed in Moreton Bay.

Almost all spat were identified as *S. glomerata* (*n* = 63) and only two lineage B and one lineage G were identified. Out of the 75 samples we collected, only nine did not amplify. These nine samples, as well as five of the samples identified as *S. glomerata* and the lineage B and G samples were then barcoded via 16S Sanger sequencing. This confirmed that identifications made via multiplex PCRs were correct. Of the nine samples for which multiplex PCRs failed, five were also unable to be identified via sequencing. This was likely due to either inadequate amounts of template DNA or the presence of DNA inhibitors which is a common issue when working with mollusc DNA [37–39], as some of the bands produced for sequencing were weak. Three were successfully identified as *S. glomerata*, while the fourth sample was identified as a species belonging to the genus *Nanostrea*. Our sequence aligned with *Nanostrea fluctigera* sequences from Japan [40], however the identification of these species was based on morphology, which we know to be unreliable [10]. Given that this species was originally described from the Red Sea [41], it is unlikely that the *Nanostrea* species identified in this study and by Hamaguchi et al., 2017 are *Nanostrea fluctigera*. We have therefore given this species the temporary name *Nanostrea* QLD sp. 1. The 16S sequence generated for this specimen has been submitted to GenBank (accession number PP116107). To our knowledge this species has not previously been documented in Moreton Bay. All other accession numbers for generated sequences can be found in “Availability of data and materials”.

### Non-destructive DNA sampling

Out of the 104 oysters collected from Noosa, only three mortalities were recorded post pallial fluid collection after six weeks. Using the *S. glomerata* and lineage G primer sets, we were able to positively identify 33 live lineage G oysters which have been retained at Bribie Island Research Centre for future spawning trials. 64 oysters were identified as *S. glomerata* and the remaining 7 samples did not amplify and were therefore unable to be identified. Despite some samples having yields less than 5 ng/ μL, including one sample which was less than 1 ng/ μL, DNA successfully amplified and subsequent identifications were able to be made. Unamplified samples were therefore likely due to the presence of DNA inhibitors, or could have been species other than *S. glomerata* or lineage G.

## Discussion

**Fig. 5:**
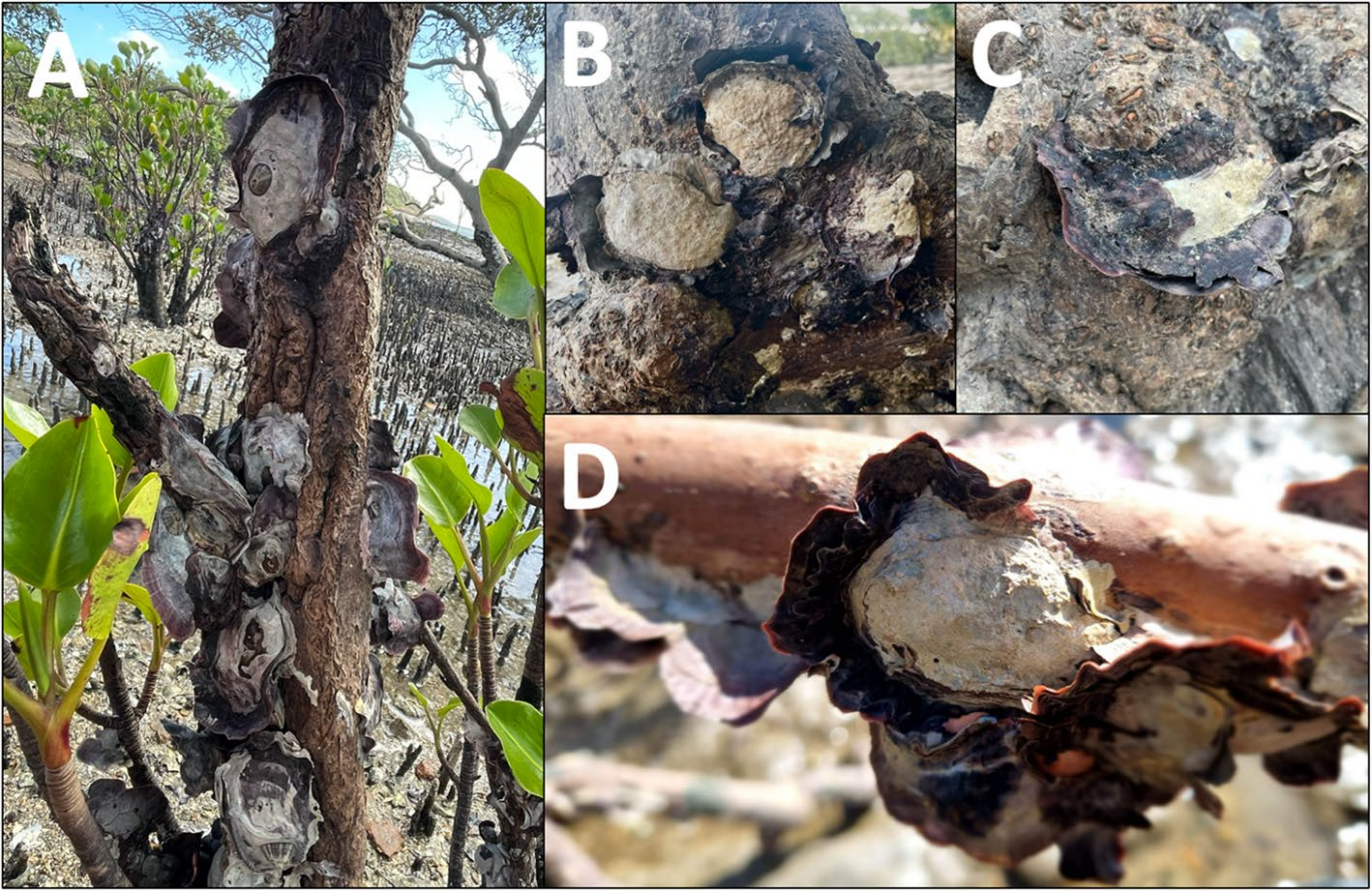
Lineage I oysters photographed on the roots and trunks of mangrove trees in Queensland, displaying characteristic broad but delicate purple lobe-like squamae (lamellae) on the lower/bottom (left) valve. **A** Gladstone; **B** and **C** Keppel Islands; and **D** Turkey Beach. Photo credit: A – C – Marina Richardson; D – Robert Porter

In this study we successfully developed a series of multiplex assays to facilitate rapid and cost-effective identification of *Saccostrea* species. Species specificity was achieved for all primer sets at an annealing temperature of 62 °C, except for the lineage B primer set which required a higher annealing temperature of 70 °C. This high temperature is not suitable for multiplex PCRs and will increase false negative lineage B detections. Using Lineage B primers in multiplex PCRs at 62 °C can be problematic, however, as they will amplify lineage I DNA and occasionally lineage G DNA. To deal with this, we recommend using the lineage B and lineage G primer sets together (along with up to two other primer sets) in a multiplex PCR, with any double bands produced at 244 bp and 380 bp identified as lineage G (see Supplementary Figure S7). Although the lineage B primer set amplified lineage I DNA at annealing temperatures below 68 °C, lineage I oysters are found almost exclusively on the aerial roots of *Rhizophora* mangroves and have several morphologically distinct features, making them one of the only species of *Saccostrea* that can be visually identified [12, 16] (Fig. 5). We therefore suggest that Lineage B primers can be used in multiplex assays (at annealing temperatures of 62 °C), so long as lineage I oysters are visually identified prior to DNA extractions, and lineage B and G primer sets are used conjunctively in PCR reactions. Given that lineage B and lineage G have closely overlapping distributions in Queensland [12], these primers should always be used together in both subtropical regions along with *S. glomerata* primers, and in tropical regions along with lineage F and *S. spathulata* primers. In tropical and subtropical Western Australia, however, all primers need to be tested for non-targeted amplification of lineage A before multiplex assays can be used for reliable identification in this region [15].

In this study we demonstrated the utility of our developed species-specific primer sets in multiplex assays in both restoration and aquaculture contexts. The results from our spat identifications in Moreton Bay showed that *S. glomerata* was the most common *Saccostrea* species at this site. Although lineage B and lineage G were also identified, they were not observed in high enough numbers to be classified as reef-building. *S. cuccullata* is often cited as a reef-building species in the tropics [22, 42, 43], however reef-building capabilities of specific lineages within this “superspecies” remains unknown. *S. glomerata* does not occur in the tropics [12], so at least one other *‘S. cuccullata*’ lineage must form reefs. Both lineage B and lineage G have widespread distributions across tropical Australia and the Indo-Pacific [7, 11], and it is possible that either (or both) of these lineages could be reef-building in these areas. The development of these multiplex assays will enable the identification of large numbers of oysters in future studies to determine this rapidly and cost-effectively.

The distributions of multiple oyster species should also be monitored to assess whether there will be range shifts in response to climate change, and what potential problems or opportunities may arise. Southern shifts into colder waters have already been documented for *S. glomerata* [44], and as the effects of climate change are intensified in subtropical regions it is possible that *S. glomerata* reefs could eventually be replaced by tropical reef-building species [45–47]. The development of these multiplex assays will be an essential tool for future monitoring as the visual misidentification of oysters could lead to the mistaken detection of range shifts. For example, there are existing records for *S. glomerata* in far north Queensland and the Indo-Pacific [22, 23], however these specimens have almost certainly been misidentified, given that surveys based on genetic identification limit *S. glomerata* to the subtropics [7, 12]. Future assessments of range shifts should consider the robustness of species records that are based on morphology alone.

**Fig. 6:**
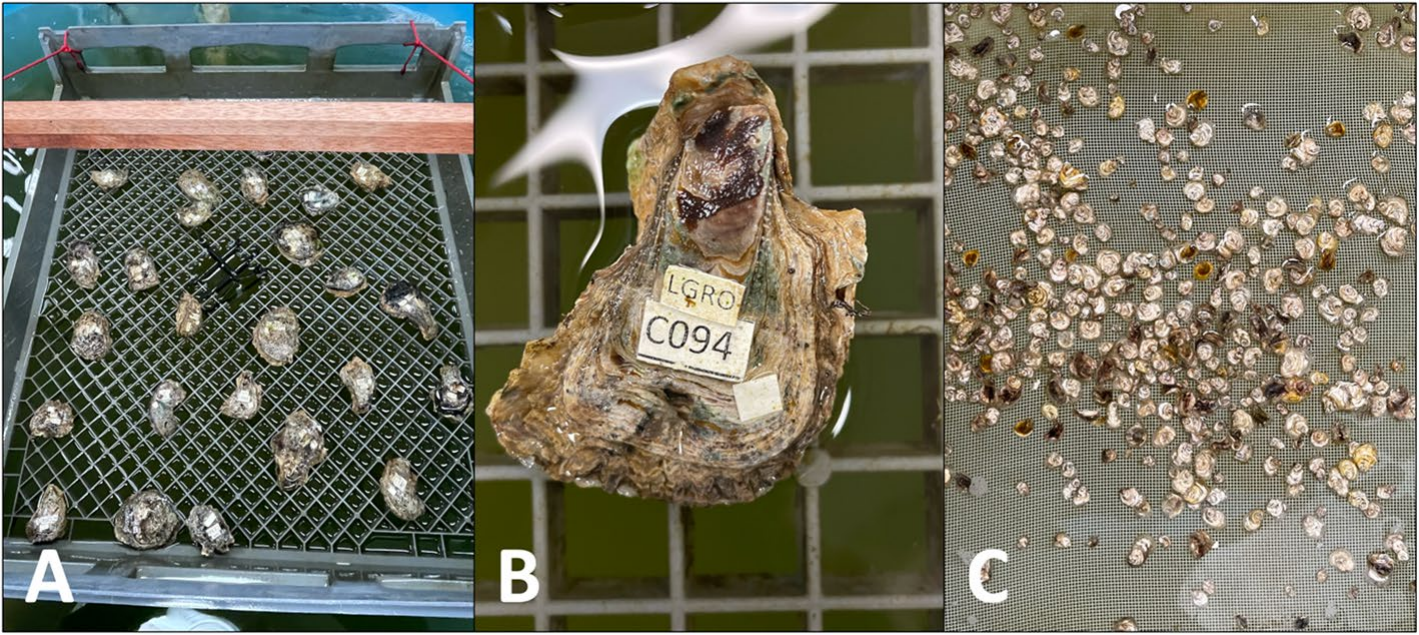
**A** Lineage G samples positively identified non-destructively; **B** a close-up image of a positively identified lineage G oyster; and **C** lineage G spat following a successful spawning trial. Photo credit: Max Wingfield

In this study we also demonstrate how the development of these multiplex assays coupled with successful non-destructive DNA sampling methods have made it possible to identify live oysters. Sanger sequencing for unambiguous identification generally requires higher DNA input for reliable reads, which can be challenging for non-destructive sampling methods where yields can be too low. In most cases we were still able to identify oysters by amplifying DNA with yields as low as 1 – 5 ng/ μL using our primer sets. The positive identification of lineage G has been a breakthrough for aquaculture, and lineage G spawning runs and grow out trials are currently underway at the Bribie Island Research Centre (Fig. 6). With no pre-existing information on the spawning or seasonality of lineage G, future research investigating the biology of the species including spawning triggers, larval development, settlement cues and substrates, growth, susceptibility to disease, potential hybridisation and preferred temperature and salinity will be essential in assessing their performance and potential for aquaculture and/or shellfish reef restoration.

The COI marker gene has become one of the most popular genes for species identifications and metabarcoding [48, 49]. Although sequencing gives more reliable results, sequencing of this region of the gene is notoriously difficult in invertebrates and especially bivalves, and often PCR bands are not obtained, or sequencing results from obtained bands are poor [37–39, 50, 51]. The multiplex primer assays designed in this study enables species identification in this eventuality, and identifications can be undertaken in a standard laboratory without the additional cost of capillary sequencing. The methods developed in this study will be most beneficial when species identifications are required for large numbers of individual specimens, however, we recommend that a subset is sequenced to confirm accuracy. The successful development of multiplex species-specific primer sets depends on the availability of diagnostic single nucleotide polymorphism [25]. Although the COI/cox1 gene is highly variable, available sequences for *Saccostrea* species and lineages are generally less than 600 bp long, making the development of multiple species-specific primers of unique product lengths challenging. The diversity of *Saccostrea* species and lineages in the Indo-Pacific is even higher than the diversity in Australia [6, 11]. Generating longer sequences for individual *Saccostrea* species and lineages occurring throughout region will therefore be instrumental to the successful development of future species-specific primers.

## Conclusion

The development of multiplex primer assays will enable the rapid, cost-effective, and accurate identification of *Saccostrea* species and lineages across tropical Australia. All tropical species and lineages of *Saccostrea* occurring in Queensland also have broad distributions across the Indo-Pacific [7, 10, 11, 40]. The assays developed in this study present easily replicable methods for the development of additional species-specific primer sets across the tropics, and future studies can therefore build on this work by testing these primers on additional *Saccostrea* species lineages found in the Indo-Pacific. Visual misidentifications and the use of incorrect species names have perpetuated a state of confusion for understanding the true diversity, ecology, and distribution of tropical oysters. Incorporating these primers into future studies will therefore ensure that species are correctly identified, aiding in the accurate collation of species-specific information to advance both restoration and aquaculture in tropical and subtropical regions.

### Supplementary Information

Supplementary Material 1.

## Data Availability

The sequences generated during this study are available at GenBank. The accession numbers are: Nanostrea sp1. QLD = PP116107; Saccostrea glomerata = PP261151, PP261152, PP261153, PP261154, PP261155, PP261156, PP261157, PP261158; Saccostrea sp. non-mordax lineage B = PP261159, PP261160; and Saccostrea sp. non-mordax lineage G = PP261161.
