# Supplementary material for: The development of multiplex PCR assays for the rapid identification of multiple Saccostrea species, and their practical applications in restoration and aquaculture
Source: BMC Ecol Evol. 2024 May 21;24:67. doi: 10.1186/s12862-024-02250-1 (PMC11107002; doi:10.1186/s12862-024-02250-1)
Supplement: Supplementary file 1 — Supplementary Material 1. [file 12862_2024_2250_MOESM1_ESM.docx]

**Supplementary Material**

**
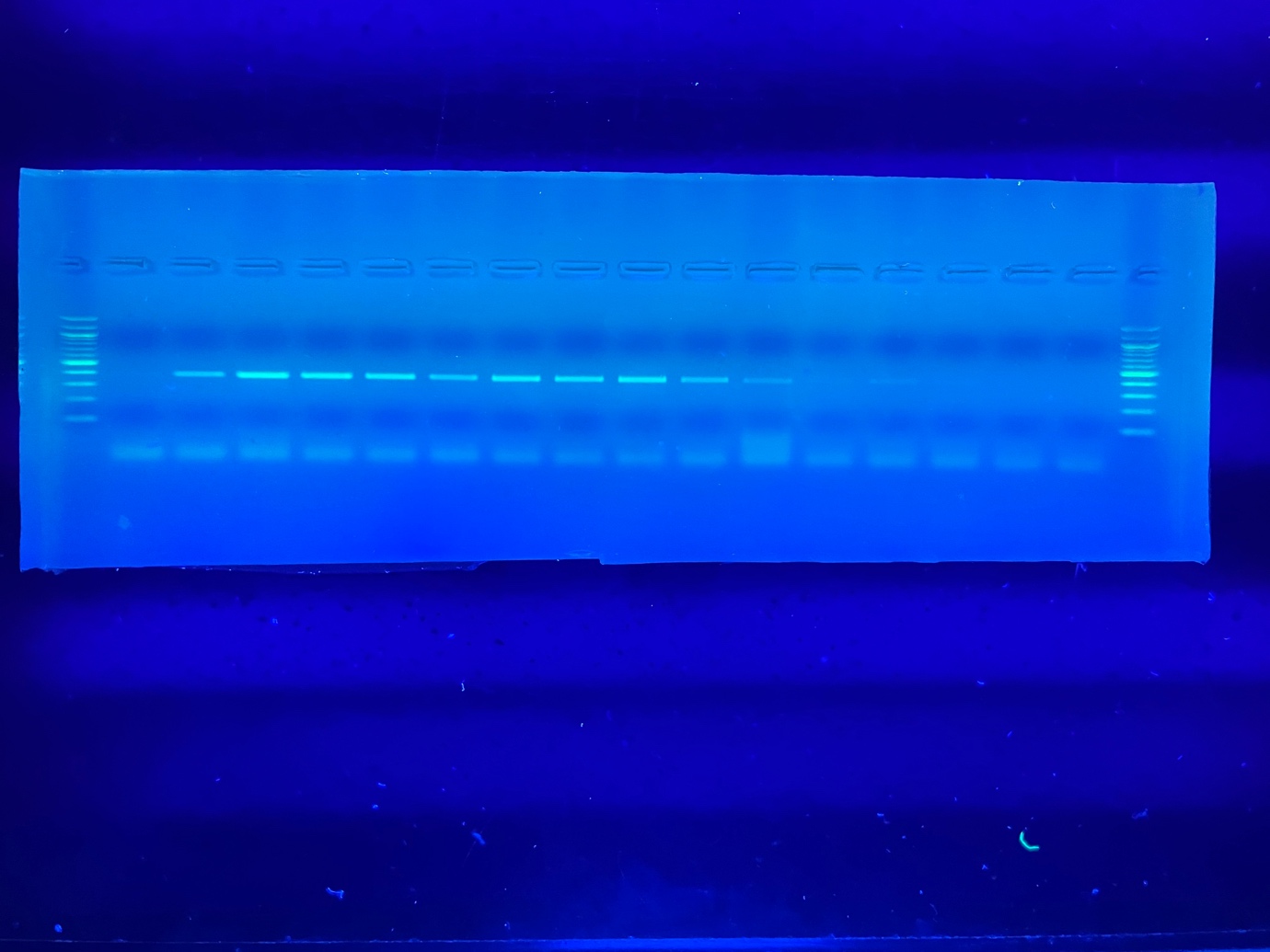
**

**Figure S1.** Original photograph of Figure 3a.


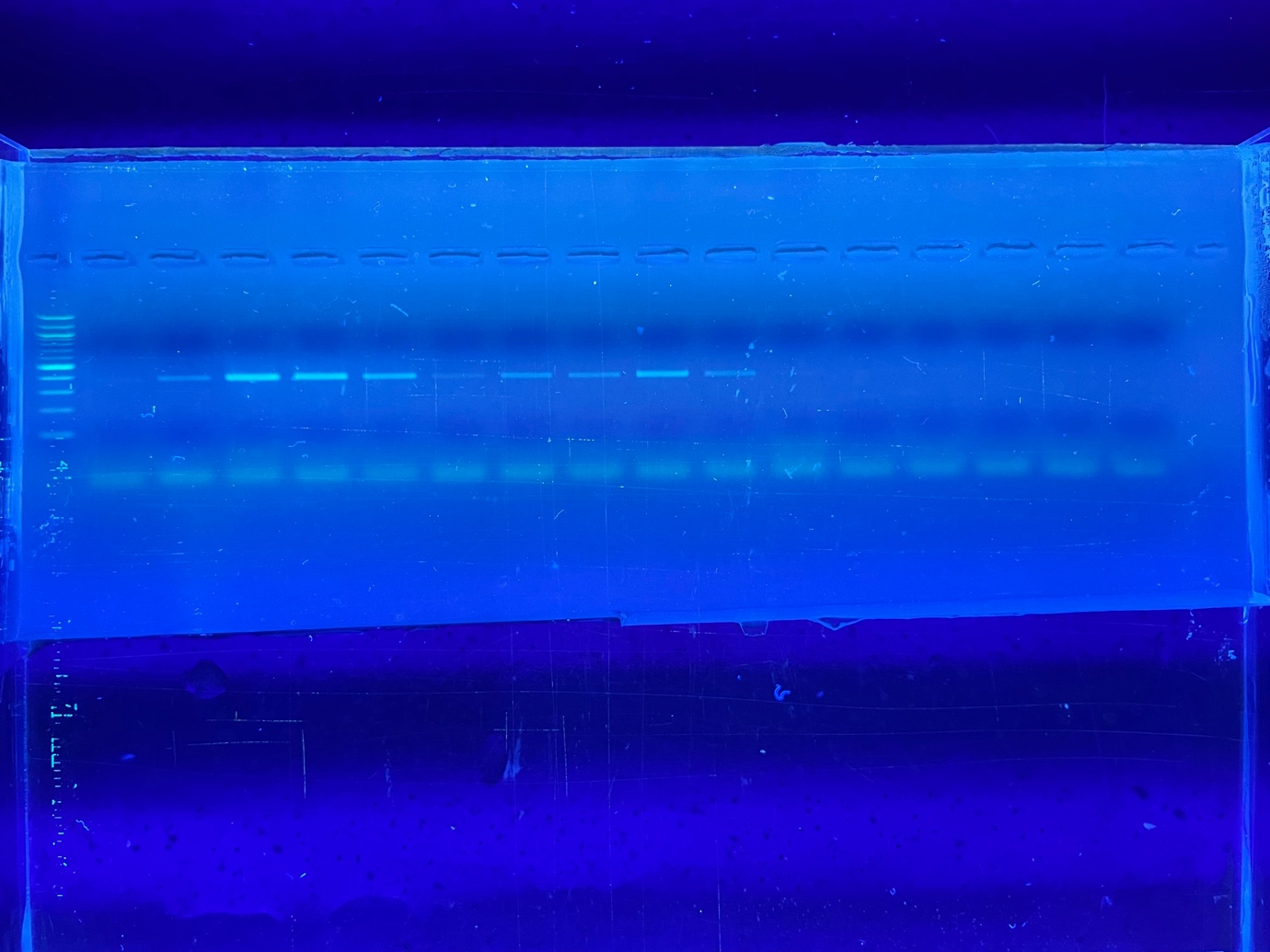


**Figure S2.** Original photograph of Figure 3b.


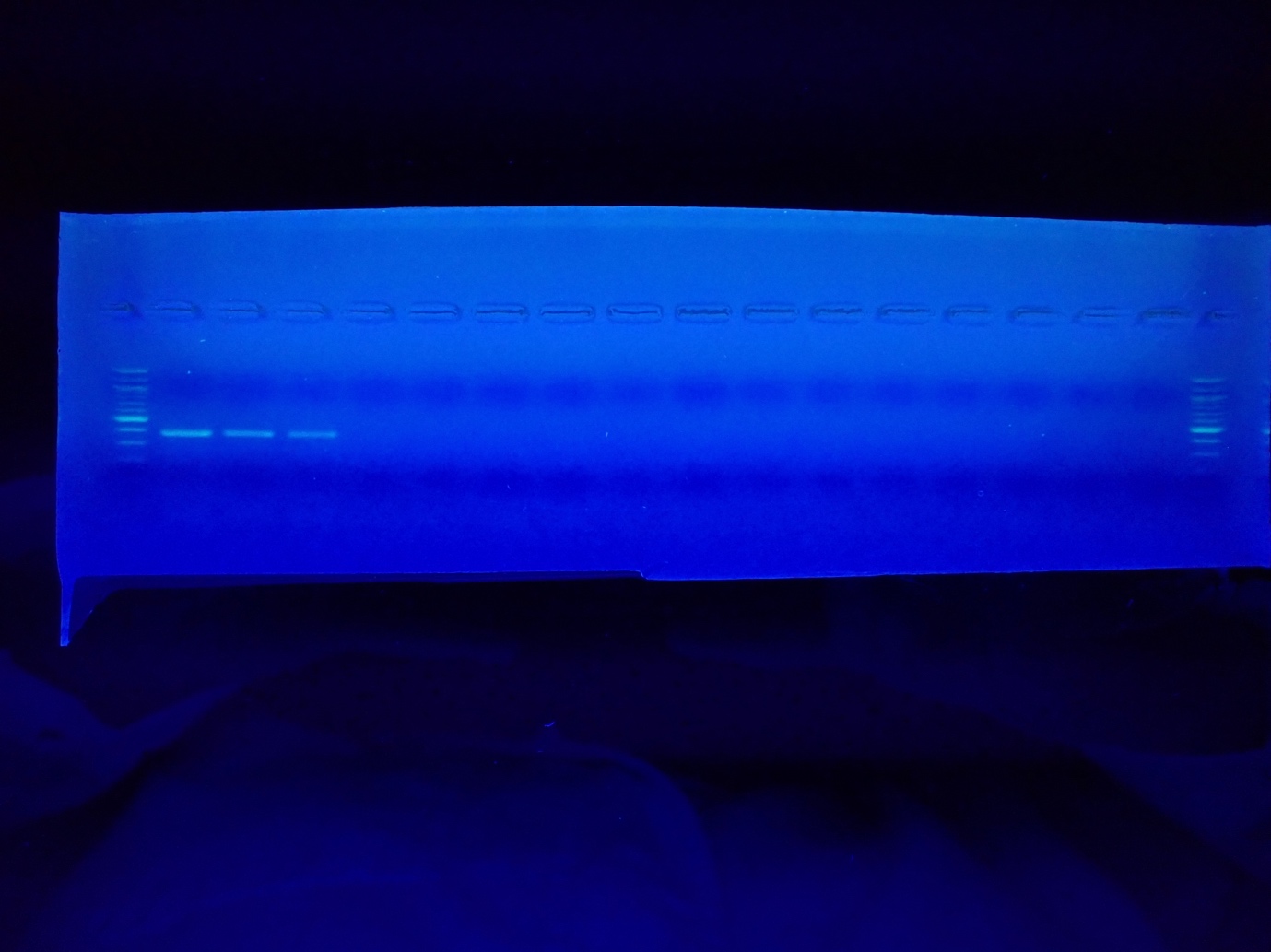


**Figure S3.** Original photograph of Figure 3c.


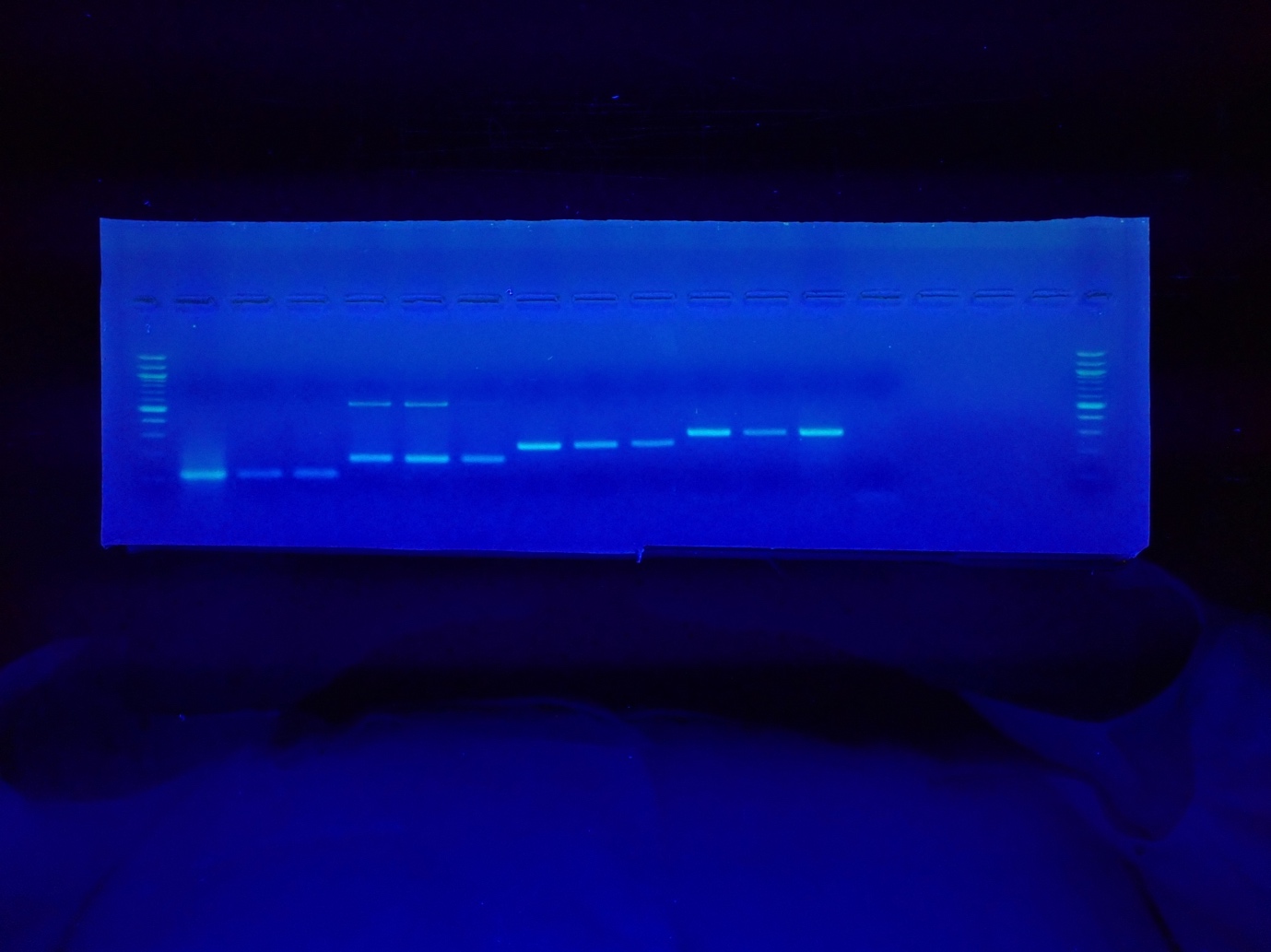


**Figure S4.** Original photograph of Figure 4a.


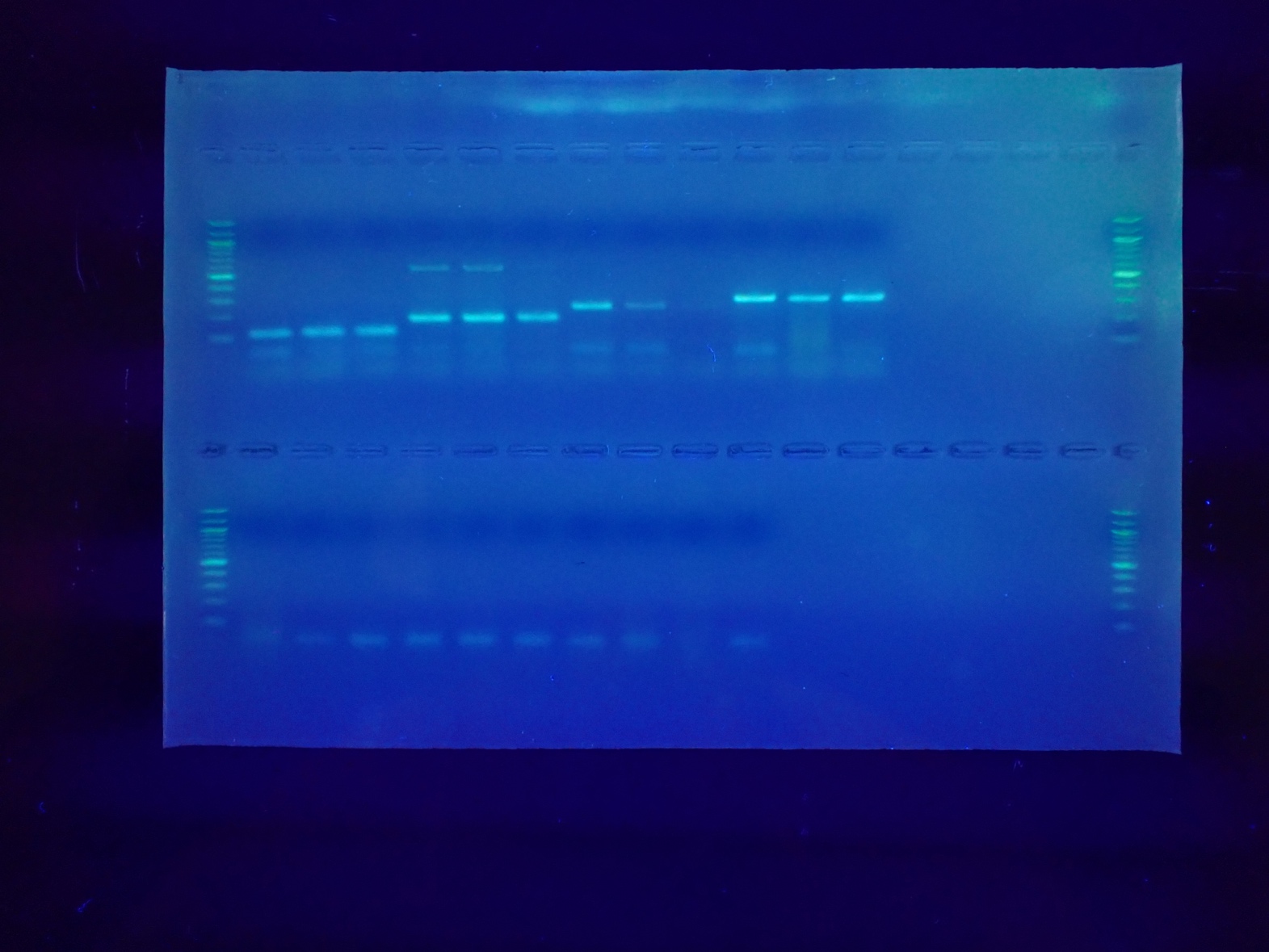


**Figure S5.** Original photograph of Figure 4b.


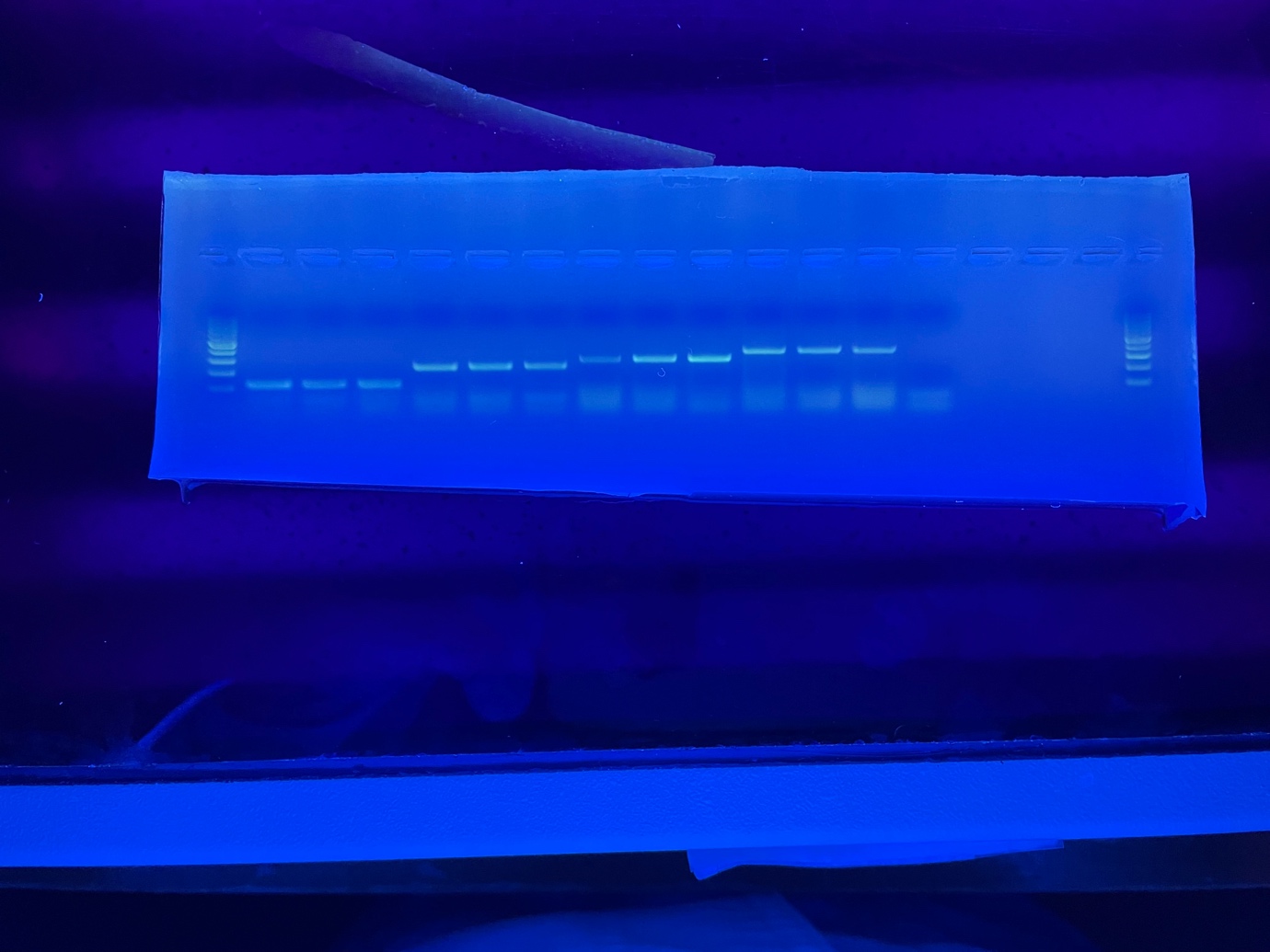


**Figure S6.** Original photograph of Figure 4c.


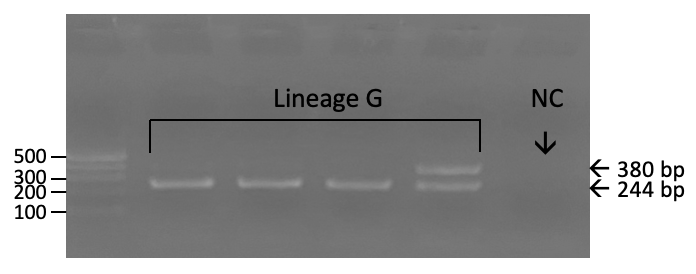


**Figure S7.** Gel electrophoresis of lineage G DNA using a multiplex PCR with lineage G (244 bp) and lineage B (380 bp) primers run at 62 °C annealing. The double band produced in lane 4 is one of the four lineage G specimens out of 55 tested samples that amplified to lineage B primers. NC = negative control.


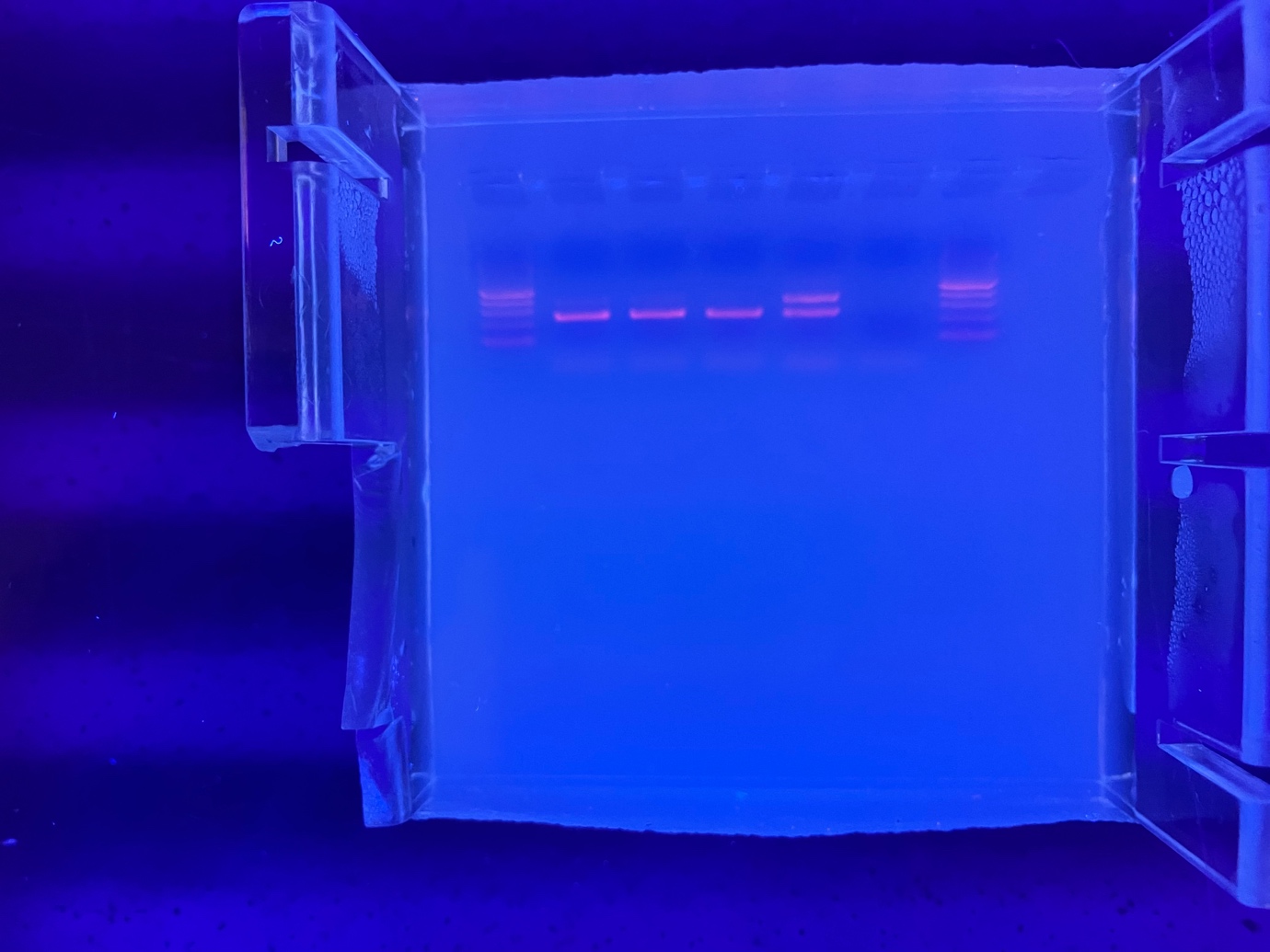


**Figure S8**. Original photograph of Figure S7.
